# Joint probabilistic-logical refinement of multiple protein feature predictors

**DOI:** 10.1186/1471-2105-15-16

**Published:** 2014-01-15

**Authors:** Stefano Teso, Andrea Passerini

**Affiliations:** 1 Department of Information Engineering and Computer Science, Università degli Studi di Trento, Trento, Italy

## Abstract

**Background:**

Computational methods for the prediction of protein features from sequence are a long-standing focus of bioinformatics. A key observation is that several protein features are closely inter-related, that is, they are conditioned on each other. Researchers invested a lot of effort into designing predictors that exploit this fact. Most existing methods leverage inter-feature constraints by including known (or predicted) correlated features as *inputs* to the predictor, thus conditioning the result.

**Results:**

By including correlated features as inputs, existing methods only rely on one side of the relation: the output feature is conditioned on the known input features. Here we show how to *jointly *improve the outputs of multiple correlated predictors by means of a probabilistic-logical consistency layer. The logical layer enforces a set of weighted first-order rules encoding biological constraints between the features, and improves the raw predictions so that they least violate the constraints. In particular, we show how to integrate three stand-alone predictors of correlated features: subcellular localization (Loctree [J Mol Biol 348:85–100, 2005]), disulfide bonding state (Disulfind [Nucleic Acids Res 34:W177–W181, 2006]), and metal bonding state (MetalDetector [Bioinformatics 24:2094–2095, 2008]), in a way that takes into account the respective strengths and weaknesses, and does not require any change to the predictors themselves. We also compare our methodology against two alternative refinement pipelines based on state-of-the-art sequential prediction methods.

**Conclusions:**

The proposed framework is able to improve the performance of the underlying predictors by removing rule violations. We show that different predictors offer complementary advantages, and our method is able to integrate them using non-trivial constraints, generating more consistent predictions. In addition, our framework is fully general, and could in principle be applied to a vast array of heterogeneous predictions without requiring any change to the underlying software. On the other hand, the alternative strategies are more specific and tend to favor one task at the expense of the others, as shown by our experimental evaluation. The ultimate goal of our framework is to seamlessly integrate full prediction suites, such as Distill [BMC Bioinformatics 7:402, 2006] and PredictProtein [Nucleic Acids Res 32:W321–W326, 2004].

## Background

Automatic assessment of protein features from amino acid sequence is a fundamental problem in bioinformatics. Reliable methods for inferring features such as secondary structure, functional residues, subcellular localization, among others, are a first step towards elucidating the function of newly sequenced proteins, and provide a complement and a reasonable alternative to difficult, expensive and time-consuming experiments. A wealth of predictors have been developed in the last thirty years for inferring many diverse types of features, see e.g. Juncker *et al.*[1] for a review.

A key observation, often used to improve the prediction performance, is that several protein features are strongly correlated, i.e., they impose constraints on each other. For instance, information about solvent accessibility of a residue can help to establish whether the residue has a functional role in binding other proteins or substrates [2], whether it affects the structural stability of the chain [3], whether it is susceptible to mutations conferring resistance to drugs [4], whether it occurs within a flexible or disordered segment [5], *etc.* There are several other examples in the literature.

Researchers have often exploited this observation by developing predictors that accept correlated features as additional inputs. This way, the output is conditioned on the known value of the input features, thus reducing the possible inconsistencies. It is often the case that the additional input features are themselves predicted. Highly complex prediction tasks like 3D protein structure prediction from sequence are typically addressed by splitting the problem into simpler subproblems (e.g., surface accessibility, secondary structure), whose predictions are integrated to produce the final output. Following this practice, multiple heterogeneous predictors have been integrated into suites (see e.g. Distill [6], SPACE [7] and PredictProtein [8]) providing predictions for a large set of protein features, from subcellular localization to secondary and tertiary structure to intrinsic disorder.

However, existing prediction architectures (with a few specific exceptions, e.g. [9] and [10]) are limited in that the output feature can’t influence a possibly mis-predicted input feature. In other words, while feature relations establish a set of *mutual* constraints, all of which should simultaneously hold, current predictors are inherently *one-way*.

Motivated by this observation, we propose a novel framework for dealing with the integration and mutual improvement of correlated predicted features. The idea is to explicitly leverage all constraints, while accounting for the fact that both the inputs, i.e., the *raw* predictions, and the constraints themselves are not necessarily correct. The refinement is carried out by a probabilistic-logical consistency layer, which takes the raw predictions as inputs and a set of weighted rules encoding the biological constraints relating the features. To implement the refiner, we use Markov Logic Networks (MLN) [11], a statistical-relational learning method able to perform statistical inference on first-order logic objects. Markov logic allows to easily define complex, rich first-order constraints, while the embedded probabilistic inference engine is able to seamlessly deal with potentially erroneous data and soft rules. We rely on an adaptation of MLN allowing to include grounding-specific weights (grounding specific Markov Logic Networks) [12], i.e. weights attached to specific instances of rules, corresponding in our setting to the raw predictions. The resulting refining layer is able to improve the raw predictions by removing inconsistencies and constraint violations.

Our method is very general. It is designed to be applicable, in principle, to any heterogeneous set of predictors, abstracting away from their differences (inference method, training dataset, performance metrics), without requiring any changes to the predictors themselves. The sole requirement is that the predictions be assigned a confidence or reliability score to drive the refinement process.

As an example application, we show how to apply our approach to the joint refinement of three highly related features predicted by the PredictProtein Suite [8]. The target features are subcellular localization, generated with Loctree [13]; disulfide bonding state, with Disulfind [14]; and metal bonding state, with MetalDetector [15].

We propose a few simple, easy to interpret rules, which represent biologically motivated constraints expressing the expected interactions between subcellular localization, disulfide and metal bonds.

The target features play a fundamental role in studying protein structure and function, and are correlated in a non-trivial manner. Most biological processes can only occur in predetermined compartments or organelles within the cell, making subcellular localization predictions an important factor for determining the biological function of uncharacterized proteins [13]; furthermore, co-localization is a necessary prerequisite for the occurrence of physical interactions between binding partners [16], to the point that lack thereof is a common mean to identify and remove spurious links from experimentally determined protein-protein interaction networks. Disulfide bridges are the result of a post-translational modification consisting in the formation of a covalent bond between distinct cysteines either in the same or in different chains [17]. The geometry of disulfide bonds is fundamental for the stabilization of the folding process and the final three-dimensional structure by fixing the configuration of local clusters of hydrophobic residues; incorrect bond formation can lead to misfolding [18]. Furthermore, specific cleavage of disulfide bonds directly controls the function of certain soluble and cell-membrane proteins [19]. Finally, metal ions provide key catalytic, regulatory or structural features of proteins; about 50% of all proteins are estimated to be metalloproteins [20], intervening in many aspects of of the cell life.

Subcellular localization and disulfide bonding state are strongly correlated: a reducing subcellular environment makes it less likely for the protein to form disulfide bridges [21]. At the two extremes we find the cytosol, which is clearly reducing, and the extra-cellular environment for secreted proteins, which is oxydizing and does not hinder disulfide bonds, with the other compartments (nucleus, mitochondrion, *etc.*) exhibiting milder behaviors. Similarly, due to physicochemical and packing constraints, it is unlikely for a cysteine to link both another cysteine (or more than one) and a ligand; with few exceptions, cysteines are involved in at most one of these bonds [15].

This is the kind of prior knowledge we will use to carry out the refinement procedure. We note that all these constraints are not *hard*: they hold for a majority of proteins, but there are exceptions [21]. In the following, we will show that that different predictors offer complementary advantages, and how our method is able to integrate them using non-trivial constraints, resulting in an overall improvement of prediction accuracy and consistency.

### Overview of the proposed method

**Figure 1:**
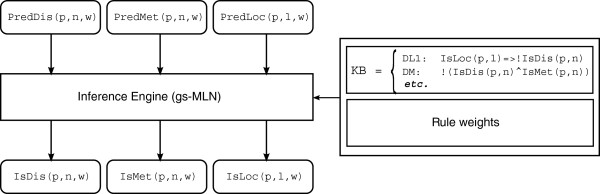
Refinement pipeline.

In this paper we propose a framework to jointly refine existing predictions according to known biological constraints. The goal is to produce novel, refined predictions from the existing ones, so as to minimize the inconsistencies, in a way that requires minimal training and no changes to the underlying predictors. The proposed system takes the raw predictions, which are assumed to be associated with a confidence score, and passes them through a probabilistic-logical consistency layer. The latter is composed of two parts: a knowledge base (KB) of biological constraints relating the features to be refined, encoded as weighted first-order logic formulae, which acts as an input to the second part of the method; and a probabilistic-logical inference engine, implemented by a grounding-specific Markov Logic Network (gs-MLN) [15]. For a graphical depiction of the proposed method see Figure 1.

An example will help to elucidate the refinement pipeline. For simplicity, let’s assume that we are interested in refining only two features: subcellular localization and disulfide bonding state. The first step is to employ two arbitrary predictors to generate the raw predictions for a given protein P. Note that disulfide bonding state is a per-cysteine binary prediction, while subcellular localization is a per-protein *n*-ary prediction; both have an associated reliability score, which can be any real number. For a complete list of predicates used in this paper, see Table 1.

**Table 1:** Predicates

**Predicate**	**Meaning**
PredLoc(p,l,w)	Protein p is predicted in compartment l with
	confidence w
PredDis(p,n,w)	Cysteine at position n is predicted disulf. bound
	with confidence w
PredMet(p,n,w)	Cysteine at position n is predicted metal bound
	with confidence w
IsLoc(p,l)	Protein p is in compartment l
IsDis(p,n)	Cysteine at position n is disulf. bound
IsMet(p,n)	Cysteine at position n is metal bound
ProxyLoc(p,l)	Proxy predicate to account for estimated Loctree
	performance
ProxyDis(p,n)	Proxy predicate to account for estimated
	Disulfind performance
ProxyMet(p,n)	Proxy predicate to account for estimated
	MetalDetector performance

Let’s assume that the predictions are as follows:

PredLoc(P,Nuc,0.1), PredLoc(P,Cyt,1.2)!PredLoc(P,Mit,0.8), PredLoc(P,Ext,1.0)!PredDis(P,11,0.2)PredDis(P,20,0.8)PredDis(P,26,0.6)

where ! stands for logical negation. The first four predicates encode the fact that protein P is predicted to reside in the nucleus with confidence 0.1, in the cytosol with confidence 1.2, *etc. *The remaining three predicates encode the predicted bonding state of three cysteines at positions 11, 20 and 26: the first cysteine is free with confidence 0.2, the remaining two are bound with confidence 0.8 and 0.6, respectively. In this particular example, the protein is assigned conflicting predictions, as the cytosolic environment is known to hinder the formation of disulfide bridges. We expect one of them to be wrong.

Given the above logical description, our goal is to infer a new set of *refined* predictions, encoded as the predicates IsLoc(p,l) and IsDis(p,n). To perform the refinement, we establish a set of logical rules describing the constraints we want to be enforced, and feed it to the inference engine. For a list of rules, see Table 2.

**Table 2:** Knowledge base

**Rule name**	**Weight**	**Rule**	**Description**
I1	per-protein	PredLoc(p,l,w) ∧ IsLoc(p,l)	Input rule for subcellular localization
I2	per-cysteine	PredDis(p,n,w) ∧ IsDis(p,n)	Input rule for disulfide bonding state
I3	per-cysteine	PredMet(p,n,w) ∧ IsMet(p,n)	Input rule for metal bonding state
I1P	per-protein	PredLoc(p,l,w) ∧ ProxyLoc(p,l)	Input rule for proxy subcellular localization
I2P	per-cysteine	PredDis(p,n,w) ∧ ProxyDis(p,n)	Input rule for proxy disulfide bonding state
I3P	per-cysteine	PredMet(p,n,w) ∧ ProxyMet(p,n)	Input rule for proxy metal bonding state
PX1	from data	ProxyLoc(p,l) ⇔ IsLoc(p,l)	Proxy rule for subcellular localization
PX2	from data	ProxyDis(p,n) ⇔ IsDis(p,n)	Proxy rule for disulfide bonding state
PX3	from data	ProxyMet(p,n) ⇔ IsMet(p,n)	Proxy rule for metal bonding state
DL1	from data	IsLoc(p,l) ⇒!IsDis(p,n)	Compartment lhinders the formation of disulf. bonds
DL2	from data	IsLoc(p,l) ⇒IsDis(p,n)	Compartment l favors the formation of disulf. bonds
DM	from data	!(IsDis(p,n) ∧IsMet(p,n))	A half-cysteine can’t bind a metal atom
L1	*∞*	$\underset{\text{l}}{\vee }$ IsLoc(p,l)	A protein must belong to at least onecompartment
L2	*∞*	∀l1IsLoc(p,l1)$\wedge \underset{\text{l2}}{\wedge }$!IsLoc(p,l2)	A protein must belong to at most onecompartment

First of all, we need to express the fact that the raw predictions should act as the primary source of information for the refined predictions. We accomplish this task using the input rules I1 and I2. These rules encode how the refined prediction predicates IsDis and IsLoc depend primarily on the raw predicates PredDis and PredLoc. The weight w is computed from the estimated reliability output by the predictor, and (roughly) determines how likely the refined predictions will resemble the raw ones.

Next we need to express the fact that a protein must belong to at least one cellular compartment, using rule L1, and, as normally assumed when performing subcellular localization prediction, that it can not belong to more than one, using rule L2. In this example, and in the rest of the paper, we will restrict the possible localizations to the nucleus, the cytosol, the mitochondrion, and the extracellular space. The two above rules are assigned an infinite weight, meaning that they will hold with certainty in the refined predictions.

The last two rules used in this example are DL1 and DL2, which express the fact that the cytosol, mitochondrion and nucleus tend to hinder the formation of disulfide bridges, while the extracellular space does not. In this case, the weights associated to the rules are inferred from the training set, and reflect how much the rules hold in the data itself.

Once we specify the raw predictions and knowledge base, we feed them to the gs-MLN. The gs-MLN is then used to infer the set of refined predictions, that is, the IsLoc and IsDis predicates. The gs-MLN allows to query for the set of predictions that is both most similar to the raw predictions, and at the same time violates the constraints the least, taking in account the confidences over the raw predictions and the constraints themselves. See the Methods section for details on how the computation is performed. In this example, the result of the computation is the following: IsLoc(P,Ext), IsDis(P,11), IsDis(P,20), IsDis(P,26). The protein is assigned to the second most likely subcellular localization, “extracellular”, and the cysteine which was predicted as free with a low confidence is changed to disulfide bonded.

It is easy to see that this framework allows to express very complicated rules between an arbitrary number of features, without particular restrictions on their type (binary, multi-label) and at different levels of detail (per-residue or per-protein). Furthermore, this approach minimizes the impact of overfitting: there is only one learned weight for each rule, and very few rules. To assess the performance of our refiner, we experiment with improving subcellular localization together with disulfide bonding state and metal bonding state. The knowledge base used for localization and disulfide bridges was introduced in this section. As for metals, the information is input using rule I3, and we model the interaction with disulfide bonds through rule DM, which states that the two types of bonds are mutually exclusive.

### Related work

There is a vast body of work dedicated to the issue of information integration, and in particular to the exploitation of correlated protein features. In many cases, the proposed methods are limited to augmenting the inputs using correlated features (either true or predicted) as additional hints to the predictors. In this setting, a work closely related to ours is [22], in which Savojardo and colleagues propose a prediction method for disulfide bridges that explicitly leverages predicted subcellular localization [23]. As in the other cases, the authors implement a one-way approach, in which a predicted feature (localization) is employed to improve a related one (disulfide bonding state). The protein prediction suites briefly mentioned above (Distill [6], SPACE [7] and PredictProtein [8]) provide another clear example of one-way architectures. Prediction suites are built by stacking multiple predictors on top of each other, with each layer making use of the predictions computed by the lower parts of the stack. In this case, the main goal is the computation of higher-level features from simpler ones. Note however that the issue of two-way consistency is ignored: these architecture do not back-propagate the outputs of the upper layers to the bottom ones. On the other hand, our approach allows to jointly improve *all* predictions by enforcing consistency in the refined outputs.

Another popular way to carry out the prediction of correlated features is multi-task learning. In this setting, one models each prediction task as a separate problem and trains all the predictors jointly. The main benefit comes from allowing information to be shared between the predictors during the training and inference stages. These methods can be grouped in two categories: iterative and collective.

Iterative methods exploit correlated predictions by re-using them as inputs to the algorithm, and iterating the training procedure until a stopping criterion is met. This approach can be found in, *e.g.* Yip *et al.*[10], which proposes a method to jointly predict protein-, domain-, and residue-level interactions between distinct proteins. Their proposal involves modeling the propensity of each protein, domain and residue to interact with other objects at the same level as a distinct regression task. After each iteration of the training/inference procedure, the most confident predictions at one level are propagated as additional training samples at the following level. This simple mechanism allows for information to bi-directionally flow between different tasks and levels. Another very relevant work is [9], in which Maes *et al.* jointly predict the state of five sequential protein features: secondary structure (in 3 and 8 states), solvent accessibility, disorder and structural alphabet. Also in this case, distinct predictors are run iteratively using the outputs at the previous time slice as additional inputs. Collective methods instead focus on building combinations of classifiers, e.g., neural network ensembles, using shared information in a single training iteration. As an example, [24] describes how to maximize the diversity between distinct neural networks with the aim of improving the overall accuracy. However most applications in biology focus on building ensembles of predictors for the *same* task, as is the case in Pollastri *et al.*[25] for secondary structure.

The main differences with our method are the following: (a) There exist a number of independently developed predictors for a plethora of correlated features. It would be clearly beneficial to refine their predictions in some way. Our goal is to be able to integrate them without requiring any change to the predictors themselves. The latter operation may be, in practice, infeasible, either because the source is unavailable, or because the cost of retraining after every change is unacceptably high. All of the methods presented here are designed for computing predictions from the ground up; our method is instead designed for this specific scenario. (b) Our method allows one to control the refinement process by including prior knowledge about the biological relationships affecting the features of interest; furthermore the language used to encode the knowledge base, first-order logic, is well defined and flexible. The other methods are more limited: any prior knowledge must be embedded implicitly in the learning algorithm itself. (c) The weights used by our algorithm are few, simple statistics of the data, and do not require any complex training. On the other hand, all the methods presented here rely on a training procedure, and have a higher risk of incurring in overfitting issues.

## Results and discussion

### Data preparation

We assessed the performance of our framework on a representative subset of the Protein Data Bank [26], the 2010/06/16 release of PDBselect [27]. The full dataset includes 4,246 unique protein chains with less than 25% mutual sequence similarity.

Focusing only on proteins containing cysteines, we extracted the true disulfide bonding state using the DSSP software [28], and the true metal bonding state from the PDB structures using a contact distance threshold of 3 Å.

Metals considered in this experiment are the same used for training MetalDetector, a total of 33 unique metal atoms and 75 molecular metals. See Passerini *et al.*[29] for more details.

Subcellular localization was recovered using the annotations in DBSubLoc [30] and UniProt [31]; we translated between PDB and UniProt IDs using the chain-level mapping described by Martin [32], dropping all proteins that could not be mapped. To increase the dataset coverage, we kept all those proteins whose true localization did not belong to any of the classes predicted by Loctree (which for animal proteins amount to cytosol, mitochondrion, nucleus and extracellular – secreted), was ambiguous or missing, and marked their localization annotation as “missing”. Loctree is also able to predict proteins in a fifth, composite class, termed “organelle”, which includes the endoplasmic reticulum, Golgi apparatus, peroxysome, lysosome, and vacuole. The chemical environment within these organelles can be vastly different, so we opted for removing them from the dataset, for simplicity.

Subcellular localization prediction requires different prediction methods for each kingdom. The preprocessing resulted in a total of 1184 animal proteins, and a statistically insignificant amount of plant and bacterial proteins; we discarded the latter two. Of the remaining proteins, 526 are annotated with a valid subcellular localization (i.e. not “missing”). The data includes 5275 cysteines, of which 2456 (46.6%) are half cysteines (i.e., involved in a disulfide bridge) and 458 (8.7%) bind metal atoms.

We also have two half cysteines that bind a metal (in protein 2K4D, chain A); we include them in the dataset as-is.

### Evaluation procedure

Each experiment was evaluated using a standard 10-fold cross-validation procedure. For each fold, we computed the rule weights over the training set, and refined the remaining protein chains using those weights. The rule weights are defined as the log-odds of the probability that a given rule holds in the data, that is, if the estimated prediction reliability output by the predictor is *r*, the weight is defined as *w *= log(*r*/(1 - *r*)). Given the weights, we refine all the raw features of proteins in the test set. If the subcellular localization for a certain protein is marked as “missing”, we use the predicted localization to perform the refinement. In this case, the refined localization is not used for computing the localization performance, and only the disulfide and metal bond refinements contribute to the fold results, in a semi-supervised fashion.

For binary classification (i.e., disulfide and metal bonding state prediction) let us denote by *T*_*p*_, *T*_*n*_, *F*_*p *_and *F*_*n *_the number of true positives, true negatives, false positives, and false negatives, respectively, and *N* the total number of instances (cysteines). We evaluate the performance of our refiner with the following standard measures:

(1) $$\begin{array}{llll} Q & = & \frac{T_{p}+T_{n}}{N} & \end{array}$$

(2) $$\begin{array}{llll} P & = & \frac{T_{p}}{T_{p}+F_{p}} & \end{array}$$

(3) $$\begin{array}{llll} R & = & \frac{T_{p}}{T_{p}+F_{n}} & \end{array}$$

(4) $$\begin{array}{llll} F_{1} & = & \frac{2\cdot P\cdot R}{P+R} & \end{array}$$

The accuracy *Q*, precision *P* and recall *R* are standard performance metrics. The *F*_1 _score is the harmonic mean of precision and recall, and is useful as an estimate balancing the contribution of the two complementary measures. We report the average and standard deviation of all above measures taken over all folds of the cross-validation.

For multi-class classification (subcellular localization) we compute the confusion matrix *M* over all classes, where each element *M*_*ij *_counts the number of instances whose true class is *i* and were predicted in class *j*. The more instances lie on the diagonal of the confusion matrix, the better the predictor.

We note that, in general, it is difficult to guarantee that our test set does not overlap with the training set of the individual raw predictors. This may result in an artificial overestimate of the performance of the raw predictors. However, training in our model consists in estimating the rule weights from the raw predictions themselves. As a consequence, the results of our refiner may be underestimated when compared with the inflated baseline performance. We also note that, since our model requires estimating very few parameters, i.e., one weight per rule, it is less susceptible to overfitting than methods having many parameters which rely on a full-blown training procedure.

### Raw predictions

We generate the predictions for subcellular localization, disulfide bridges, metal bonds and solvent accessibility using the respective predictors. All predictors were installed locally, using the packages available from the PredictProtein Debian package repository [33], and configured to use the default parameters. For all protein chains predicted in the “organelle” class, we marked the prediction as “missing”, for the reasons mentioned above.

For Disulfind and MetalDetector, we converted the per-cysteine weighted binary predictions into two binary predicates for each cysteine, PredDis/3 and PredMet/3, using as prediction confidence w the SVM margin.

For Loctree, we output four PredLoc/3 predicates for each protein, one for each possible subcellular localization, and computed the confidence by using a continuous version of the Loctree-provided output-to-confidence mapping. The raw predictor performance can be found alongside with the refiner performance in Tables 3, 4, 5, 6.

**Table 3:** Results for true Sub. Loc.

**Disulfide bonding state**				
**Experiment**	***Q***	***P***	***R***	***F***_**1**_
Raw predictions	0.804 ± 0.03	0.720 ± 0.06	0.917 ± 0.04	0.811 ± 0.04
Dis. + Met.	0.832 ± 0.04	0.767 ± 0.05	0.913 ± 0.04	0.833 ± 0.04
Dis. + Loc.	0.857 ± 0.03	0.801 ± 0.04	0.921 ± 0.03	0.856 ± 0.03
Dis. + Met. + Loc.	0.867 ± 0.03	0.819 ± 0.04	0.919 ± 0.03	0.865 ± 0.03
HMSVM	0.874 ± 0.03	0.884 ± 0.06	0.851 ± 0.03	0.866 ± 0.03
BRNN	0.892 ± 0.02	0.900 ± 0.03	0.863 ± 0.05	0.880 ± 0.03
**Metal bonding state**				
**Experiment**	*Q*	*P*	*R*	*F*_1_
Raw predictions	0.952 ± 0.02	0.686 ± 0.09	0.827 ± 0.10	0.747 ± 0.09
Dis. + Met.	0.950 ± 0.02	0.711 ± 0.09	0.739 ± 0.14	0.713 ± 0.09
Dis. + Loc.	–	–	–	–
Dis. + Met. + Loc.	0.952 ± 0.02	0.709 ± 0.08	0.783 ± 0.11	0.736 ± 0.07
HMSVM	0.950 ± 0.02	0.74 1± 0.12	0.697 ± 0.08	0.711 ± 0.07
BRNN	0.948 ± 0.02	0.683 ± 0.09	0.763 ± 0.11	0.715 ± 0.07

**Table 4:** Results for predicted Sub. Loc.

**Disulfide bonding state**				
**Experiment**	***Q***	***P***	***R***	***F***_**1**_
Raw predictions	0.804 ± 0.03	0.720 ± 0.06	0.917 ± 0.04	0.811 ± 0.04
Dis. + Met.	0.832 ± 0.04	0.767 ± 0.05	0.913 ± 0.04	0.833 ± 0.04
Dis. + Loc.	0.809 ± 0.03	0.732 ± 0.06	0.923 ± 0.04	0.815 ± 0.04
Dis. + Met. + Loc.	0.843 ± 0.03	0.779 ± 0.04	0.919 ± 0.04	0.843 ± 0.03
HMSVM	0.882 ± 0.03	0.890 ± 0.05	0.856 ± 0.04	0.872 ± 0.04
BRNN	0.884 ± 0.03	0.895 ± 0.03	0.847 ± 0.05	0.870 ± 0.03
**Metal bonding state**				
**Experiment**	*Q*	*P*	*R*	*F*_1_
Raw predictions	0.952 ± 0.02	0.686 ± 0.09	0.827 ± 0.10	0.747 ± 0.09
Dis. + Met.	0.950 ± 0.02	0.711 ±0.09	0.739 ± 0.14	0.713 ± 0.09
Dis. + Loc.	–	–	–	–
Dis. + Met. + Loc.	0.949 ± 0.02	0.707 ± 0.09	0.731 ± 0.14	0.705 ± 0.08
HMSVM	0.952 ± 0.02	0.755 ± 0.07	0.707 ± 0.09	0.725 ± 0.06
BRNN	0.950 ± 0.02	0.694 ± 0.09	0.768 ± 0.11	0.723 ± 0.08
**Subcellular localization**				
Raw predictions				
	Cytosol	ExtraCell.	Mitoch.	Nucleus
Cytosol	14	11	2	5
ExtraCell.	17	206	2	29
Mitoch.	12	8	6	8
Nucleus	46	67	15	78
Dis. + Loc.				
Cytosol	15	10	2	5
ExtraCell.	18	223	2	11
Mitoch.	12	223	2	8
Nucleus	48	55	16	87
Dis. + Met. + Loc.				
Cytosol	15	9	2	6
ExtraCell.	18	223	2	11
Mitoch.	12	6	8	8
Nucleus	48	44	21	93

**Table 5:** Results for true Sub. Loc. with proxy

**Disulfide bonding state**				
**Experiment**	***Q***	***P***	***R***	***F***_**1**_
Raw predictions	0.804 ± 0.03	0.720 ± 0.06	0.917 ± 0.04	0.811 ± 0.04
Dis. + Met.	0.838 ± 0.03	0.776 ± 0.04	0.912 ± 0.04	0.838 ± 0.04
Dis. + Loc.	0.853 ± 0.03	0.796 ± 0.05	0.921 ± 0.03	0.853 ± 0.03
Dis. + Met. + Loc.	0.865 ± 0.03	0.817 ± 0.04	0.917 ± 0.03	0.863 ± 0.03
HMSVM	0.874 ± 0.03	0.884 ± 0.06	0.851 ± 0.03	0.866 ± 0.03
BRNN	0.892 ± 0.02	0.900 ± 0.03	0.863 ± 0.05	0.880 ± 0.03
**Metal bonding state**				
**Experiment**	*Q*	*P*	*R*	*F*_1_
Raw predictions	0.952 ± 0.02	0.686 ± 0.09	0.827 ± 0.10	0.747 ± 0.09
Dis. + Met.	0.952 ± 0.02	0.696 ± 0.08	0.795 ± 0.11	0.739 ± 0.08
Dis. + Loc.	–	–	–	–
Dis. + Met. + Loc.	0.952 ± 0.02	0.695 ± 0.08	0.807 ± 0.10	0.743 ± 0.08
HMSVM	0.950 ± 0.02	0.741 ± 0.12	0.697 ± 0.08	0.711 ± 0.07
BRNN	0.948 ± 0.02	0.683 ± 0.09	0.763 ± 0.11	0.715 ± 0.07

**Table 6:** Results for predicted Sub. Loc. with proxy

**Disulfide bonding state**				
**Experiment**	***Q***	***P***	***R***	***F***_**1**_
Raw predictions	0.804 ± 0.03	0.720 ± 0.06	0.917 ± 0.04	0.811 ± 0.04
Dis. + Met.	0.838 ± 0.03	0.776 ± 0.04	0.912 ± 0.04	0.838 ± 0.04
Dis. + Loc.	0.803 ± 0.03	0.727 ± 0.05	0.922 ± 0.04	0.811 ± 0.04
Dis. + Met. + Loc.	0.846 ± 0.03	0.784 ± 0.04	0.918 ± 0.04	0.845 ± 0.04
HMSVM	0.882 ± 0.03	0.890 ± 0.05	0.856 ± 0.04	0.872 ± 0.04
BRNN	0.884 ± 0.03	0.895 ± 0.03	0.847 ± 0.05	0.870 ± 0.03
**Metal bonding state**				
**Experiment**	*Q*	*P*	*R*	*F*_1_
Raw predictions	0.952 ± 0.02	0.686 ± 0.09	0.827 ± 0.10	0.747 ± 0.09
Dis. + Met.	0.952 ± 0.02	0.696 ± 0.08	0.795 ± 0.11	0.739 ± 0.08
Dis. + Loc.	–	–	–	–
Dis. + Met. + Loc.	0.952 ± 0.02	0.706 ± 0.08	0.782 ± 0.10	0.735 ± 0.06
HMSVM	0.952 ± 0.02	0.755 ± 0.07	0.707 ± 0.09	0.725 ± 0.06
BRNN	0.950 ± 0.02	0.694 ± 0.09	0.768 ± 0.11	0.723 ± 0.08
**Subcellular localization**				
Raw predictions				
	Cytosol	ExtraCell.	Mitoch.	Nucleus
Cytosol	14	11	2	5
ExtraCell.	17	206	2	29
Mitoch.	12	8	6	8
Nucleus	46	67	15	78
Dis. + Loc.				
Cytosol	13	13	2	4
ExtraCell.	22	224	0	8
Mitoch.	12	6	8	8
Nucleus	61	56	14	75
Dis. + Met. + Loc.				
Cytosol	14	12	2	4
ExtraCell.	22	224	0	8
Mitoch.	12	6	8	8
Nucleus	67	41	17	81

### Alternative refinement pipelines

In order to assess the performance of our method, we carried out comparative experiments using two alternative refinement architectures. Both architectures are based on state-of-the-art sequential prediction methods, namely Hidden Markov Support Vector Machines (HMSVM) [34] and Bidirectional Recurrent Neural Networks (BRNN) [35]. Both methods can naturally perform classification over sequences, and have been successfully applied to several biological prediction tasks.

The alternative architectures are framed as follows. The predictors are trained to learn a mapping between raw predictions and the ground truth, using the same kind of pre-processing as the MLN refiner. Cysteines belonging to a protein chain form a single example, and all cysteines in an example are refined concurrently. The input consists of all three raw predictions in both cases.

The two methods were chosen as to validate the behavior of more standard refinement pipelines relying on both hard and soft constraints. In the case of HMSVMs, the model outputs a single label for each residue: a cysteine can be either free, bound to another cysteine, or bound to a metal. This encoding acts as a hard constraint on the mutual exclusivity between the two labels. In the case of BRNNs, each cysteine is modeled by two independent outputs, so that all four configurations (free, disulfide bound, metal bound, or both) are possible. The BRNN is given the freedom to learn the (soft) mutual exclusivity constraint between the two features from the data itself.

Pure sequential prediction methods, like HMSVMs, are at the same specialized for, and limited to, refining sequential features, in our case disulfide and metal bonding state. Therefore, we can’t use the HMSVM pipeline for localization refinement. As a result, the alternative pipeline is faced with a reduced, and easier, computational task. While BRNN are also restricted to sequential features, more general recursive neural networks [36] can in principle model arbitrary network topologies. However, they cannot explicitly incorporate constraints between the outputs, which is crucial in order to gain mutual improvement between subcellular localization and bonding state predictions. As experimental results will show, these alternative approaches already fail to jointly improve sequential labeling tasks.

We performed a 10-fold inner cross-validation to estimate the model hyperparameters (regularization tradeoff for the HMSVM, learning rate for the neural network), using the same fold splits as the main experiment. The results can be found in Table 3 through 6.

### True subcellular localization

As a first experiment, we evaluate the effects of using the true subcellular localization to refine the remaining predictions, i.e., we supply the refiner with the correct IsLoc directly, while querying the IsDis and IsMet predicates. The experiment represents the ideal case of a perfect subcellular localization predictor, and we can afford to unconditionally trust its output.

The experiment is split in four parts of increasing complexity. 

• In the ‘Dis. + Met.’ case we refine both IsDis and IsMet from the respective raw predictions, using only the DM rule (see Table 2) to coordinate disulfide and metal bonding states; the localization in this case is ignored. The experiment is designed to evaluate wheter combining only disulfide and metal predictions is actually useful in our dataset.

• In the ‘Dis. + Loc.’ case we refine IsDis from the raw disulfide predictions and the true localization, using the DL1 and DL2 rules.

• In the ‘Dis. + Met. + Loc.’ case we refine IsDis and IsMet making the refined disulfide bonding state interact with metals (using the DM rule), solvent accessibility (with the DA rule), and subcellular localization (with DL1 and DL2.)

The results can be found in Table 3.

Three trends are apparent in the results. First of all, we find subcellular localization to have a very strong influence on disulfide bonding state, as expected. In particular, in the ‘Dis. + Loc.’ case, which includes no metal predictions, the accuracy and *F*_1_ measure improves from 0.804 and 0.811 (raw) to 0.857 and 0.856 (refined), respectively. The change comes mainly from an increase in precision: the true subcellular localization helps reducing the number of false positives.

The interaction between metals and disulfide bonds is not as clear cut: in the ‘Dis. + Met.’ case, which includes no subcellular localization, the refined disulfide predictions slightly improve, in terms of *F*_1_ measure, while the metal predictions slightly worsen. The latter case is mainly due to the drop in recall, from 0.827 to 0.739. This is to be expected, as the natural scarcity of metal residues makes the metal prediction task harder (as can be seen observing the differential behavior of accuracy and *F*_1_ measure). As a consequence the confidence output by MetalDetector is lower than the confidence output by Disulfind. In other words, in the case of conflicting raw predictions, the disulfide predictions usually dominate the metal predictions.

Finally, in ‘Dis. + Met. + Loc.’ case, both disulfide and metal bonds improve using the true subcellular localization compared to the above settings. In particular, metal ligand prediction, while still slightly worse than the baseline (again, due to class unbalance, as mentioned above) sees a clear gain in recall (from 0.739 in the ‘Dis. + Met.’ case to 0.783). This is an effect of using localization: removing false disulfide positives leads to less spurious conflicts with the metals.

The two alternative pipelines behave similarly. They both manage to beat the Markov Logic Network on the easier of the two tasks, disulfide refinement, while performing worse on the metals. We note that the HMSVM and BRNN, contrary to our method, both have a chance to rebalance the raw metal predictions with respect to the disulfide predictions during the training stage, learning a distinct bias/weight for the inputs. Nevertheless, they still fail to improve upon our refined metals.

### Predicted subcellular localization

This experiment is identical to the previous one, except we use predicted subcellular localization in place of the true one. Similarly to the previous section, we consider three sub-cases. In the ‘Dis. + Loc.’ case, we refine localization and disulfide bonding state, while in the ‘Dis. + Met. + Loc.’ case we refine all three predicted features together. The results can be found in Table 4. The ‘Dis. + Met.’ case is reported as well for ease of comparison.

Here we can see how our architecture can really help with the mutual integration of protein features. In general, we notice that refined disulfide bonds are enhanced by the integration of localization, even if less so than in the previous experiment. At the same time, localization also benefits by the interaction with disulfide bonds, as can be seen in the ‘Dis. + Loc.’ case. The biggest gain is obtained for the ExtraCellular and Nucleus classes, which are also the most numerous classes in the dataset: several protein chains are moved back to their correct class. The introduction of metals improves directly disulfide bonds and indirectly localization, even though its effect is relatively minor.

On the downside, refined metal predictions worsen in all cases. This is due, again, to the unbalance of the small number of metal binding residues found in the data, and to the difference between the confidences output by Disulfind and MetalDetector.

Surprisingly, the alternative pipelines are not as affected by the worsening of the localization information: their performance is on par as with the true localization. This is in part explained by the simpler task the alternative methods carry out, as it does not involve refinement of the raw localization itself. It turns out that using predicted localization itself, the alternative methods manage to perform better than us also for metal refinement. In the following, we will show an improvement to our pipeline to address this issue.

### Predicted subcellular localization with predictor reliability

The previous experiment shows that our refiner performs suboptimally on the metal refinement task due to class unbalance. A common way to alleviate this issue is to re-weight the classes according to some criterion. In our case, the positive metal residues are dominated by the negative ones, making the overall accuracy of MetalDetector higher than that of Disulfind. Our method naturally supports the re-weighting of predictors with different accuracy: the weight assigned to a Pred predicate can be strengthened or weakened depending on our estimate of the predictor accuracy.

To implement this strategy, we add an intermediate *proxy* predicate, weighted according to the actual predictor performance over the training set. The proxy predicate mediates the interaction between the raw prediction (the Pred predicate) and the refined prediction (the Is predicate). The actual proxy predicates are ProxyLoc, ProxyDis and ProxyMet, used by rules I1P to I3P, and PX1 to PX3. See Tables 1 and 2 for the details. The results can be found in Table 6. For completeness, we also include the proxy results for true subcellular localization in Table 5.

The proxy helps the MLN refiner: the refined metal predictions are on-par with the raw ones, while at the same time improving the disulfide bonds. The effects are especially clear when comparing the ‘Dis. + Met.’ cases of Tables 3 (true localization, no proxy) and 5 (true localization, with proxy), with *F*_1_ scores changing from 0.833 and 0.713 for bridges and metals, respectively, to 0.838 and 0.739. We note that our method is the only one able to recover the same performance as MetalDetector while also improving the other two refined features. On the contrary, the alternative pipelines tend to favor one task (disulfide bridges) over the other, and fail in all cases to replicate the baseline performance.

The down-side is that localization refinement is slightly worse: the raw Nucleus predictions are less accurate than the Cytosol ones, leading to the Cytosol being assigned a higher proxy weight. Since both compartments prevent disulfide bonds, the MLN refiner tends to assign chains with no half cysteines to the latter.

## Conclusions

In this paper we introduced a novel framework for the joint integration and refinement of multiple related protein features. The method works by resolving conflicts with respect to a set of user-provided, biologically motivated constraints relating the various features. The underlying inference engine, implemented as a grounding-specific Markov Logic Network [12], allows to perform probabilistic reasoning over rich first-order logic rules. The designer has complete control over the refinement procedure, while the inference engine accounts for potential data noise and rule fallacy.

As an example, we demonstrate the usefulness of our framework on three distinct predicted features: subcellular localization, disulfide bonding state, metal bonding state. Our refiner is able to improve the predictions by removing violations to the constraints, leading to more consistent results. In particular, we found that subcellular localization plays a central role in determining the state of potential disulfide bridges, confirming the observations of Savojardo *et al.*[22]. Our method however also allows to improve subcellular localization in the process, helping to discriminate between chains residing in reducing and oxydizing cellular compartments, especially nuclear and secreted chains. We also found that disulfide predictions benefit from metal bonding information, although to a lesser extent, especially when used in conjunction with localization predictions. On the other hand metals, which are in direct competition with the more abundant disulfide bonds, are harder to refine. We presented a simple and natural re-weighting strategy to alleviate this issue. The task would be further helped by better localization predictions, which tend to improve the distribution of disulfide bridges, as shown by the experiments with true subcellular localization.

We compared our refinement pipeline with two alternatives based on state-of-the-art sequential prediction methods, Hidden Markov Support Vector Machines and Bidirectional Recursive Neural Networks. These methods have two fundamental advantages: they are run through a full-blown training procedure, and are only asked to refine the two sequential features, a task for which they are highly specialized. However, the results show that they tend to favor the easier task (disulfide bridges) over the other, struggling to achieve the same results of the baseline on the harder task (metals). On the contrary, our method is more general, and does not favor one task at the expense of the others.

Our framework is designed to be very general, with the goal of refining arbitrary sets of existing predictors for correlated features, such as Distill [6] and PredictProtein [8], for which re-training is difficult or infeasible. As a consequence, our framework does not require any change to the underlying predictors themselves, only requiring that they provide an estimated reliability for their predictions.

## Methods

### Predictors

Disulfind [14] is a web server for the prediction of disulfide bonding state and binding geometry from sequence alone. Like other tools for the same problem, Disulfind splits the task in two simpler sub-problems as follows. First an SVM binary classifier is employed to independently infer the bonding state of each cysteine in the input chain. The SVM is provided with both local and global information. Local information includes a window of position-specific conservations derived from multiple alignment, centered around each target cysteine. Global information represent global features of the whole chain, such as length, amino acid composition, and average cysteine conservation. Then a bidirectional recursive neural network (BRNN) is used to collectively refine the possibly incorrect SVM predictions, assigning a revised binding probability to each cysteine.

Finally, the predictions are post-processed with a simple finite-state automaton to enforce an even number of positive disulfide bonds. For the technical details, see Vullo *et al.*[37].

MetalDetector [29] is a metal bonding state classifier, whose architecture is very similar to Disulfind. It is split in two stages, an SVM classifier for local, independent per-residue

Loctree [13] is a multiclass subcellular localization predictor based on a binary decision tree of SVM nodes. The topology of the tree mimics the biological structure of the cellular protein sorting system. It is designed to predict the subcellular localization of proteins given only their sequence, and uses multiple input features: a multiple alignment step is performed against a local, reduced redundancy database of UniProt proteins, and makes use of a stripped, specially tailored version of Gene Ontology vocabulary terms to improve its performance. It also uses psort 3.0 [38]. The predictor incorporates three distinct topologies, one for each of the considered kingdoms: prokaryotes, eukariotic plants (viridiplantae), eukariotic non-plants (metazoa).

### First-order logic background

For the purpose of this paper, first-order logic *formulae* are used to construct a relational representation of the features of interest, their mutual constraints, and to perform probabilistic-logical reasoning on them. Some definitions are in order.

A formula can be constructed out of four syntactical classes: *constants*, which represent fixed objects in the domain (e.g., “PDB1A1IA”); *variables*, which are placeholders for constants (e.g., “protein”); *functions*, which map a tuple of objects to another object (not needed in our case); and *predicates*, which describe properties of objects (e.g., “IsDis(p,n)”), or relations between objects. Constants, variables are *terms*, and so are predicates applied to a tuple of terms. If a term contains no variables, it is said to be *ground*.

Predicates are assigned a truth value (True or False) which specifies whether the property/relation is observed to hold or not. An *atom* is a predicate applied to a tuple of terms. A *formula* is recursively defined as being either an atom, or as a set of formulae combined through logical connectives (negation !, conjunction ∧, disjunction ∨, implication ⇒, and equivalence ⇔) or quantifiers (existential ∃ or universal ∀). A formula F containing a reference to a variable x can be used to build ∀x.F, which is true iff F is true for all possible values of x in the domain, and ∃x.F, which is true iff F is true for at least one value of x. A formula F whose variables have all been replaced by constants is called a *grounding* of F.

An *interpretation* or (possible) *world* is an assignment of truth values to all ground atoms. A collection of implicitly conjoined formulae KB = $\underset{i}{\wedge }$F_*i*_ is a knowledge base, and can be seen as a single big formula. *Logical inference* is the problem of determining whether a knowledge base KB entails a given formula Q, written KB ⊧Q, which is equivalent to asking whether the formula Q is true in every interpretation (world) where KB is true.

Whenever any two formulae in a KB are in contradiction, the knowledge base admits no interpretation at all. This is an issue when reasoning over conflicting facts taken from unreliable information sources, as is often the case for biological information.

### Grounding-specific Markov logic networks

A Markov Logic network (MLN) [11], is a method to define a probability distribution over all *possible worlds* (truth assignments) of a set of formulae allowing to perform reasoning over possibly wrong or conflicting facts.

A MLN consists of a *finite* domain of objects (constants) C and a knowledge base KB of logical rules. Each formula F_*i*_ in KB is associated a real-valued weight *w*_*i*_, representing the confidence we have in that rule. Weights close to zero mean that the formula is very uncertain, while larger weights mean that it is likely to hold (if positive) or not (if negative). Contrarily to pure FOL, in Markov Logic the formulae in the KB are explicitly fallible; as a consequence, Markov Logic admits interpretations that don’t satisfy all the constraints.

Instantiating all the formulae in KB using all possible combinations of constants in C leads to a *grounding* of the knowledge base. As an example, if C consists of three objects, a protein P and two cysteines at position 4 and 19, and the knowledge base consists of the formula DM = !(IsDis(p,n) ∧IsMet(p,n)), then the grounding will be the set of ground formulae: {DM(P,4),DM(P,19)}. A *possible world* is a truth assignment of the grounding of KB. Markov Logic defines a way to assign to each possible world a probability, determined by the weight of the formulas that it satisfies.

A MLN defines a joint probability distribution over the set of interpretations (i.e. truth assignments) of the grounding of KB. In the previous example, if the formula DM has a positive weight, then the assignment DM(P,4)∧DM(P,19) will be the most likely, while ! DM(P,4)∧!DM(P,19) will be the least likely, with the other possible worlds standing in between. In addition, if an assignment satisfies a formula with a negative weight, it becomes less likely.

Given a set of ground atoms x of known state, and a set of atoms y whose state we want to determine, we can define the conditional distribution generated by a MLN as follows:

$$p(\text{y}\;|\;\text{x}\;;\;w)=\frac{1}{Z(\text{x})}\text{exp}\underset{{\text{F}}_{i}\in \text{KB}}{\sum }w_{i}n_{i}(\text{x},\text{y})$$

Here *n*_*i*_(x,y) counts how many times the formula F_*i*_ is satisfied by groundings of world (x,y), and *Z*(x) is a normalization term. In other words, the above formula says that the probability of y being in a given state is proportional to the weighted number of formulae in KB that the interpretation (x, y) satisfies. We can query a MLN for the most likely state of the unknown predicates y from the known facts x by taking the truth assignment of y that maximizes the above conditional probability. See Richardson *et al.*[11] for a full-length description.

An issue with standard Markov Logic is that distinct groundings of the same formula F_*i*_ are assigned the same weight w_*i*_. This is not the case for our raw predictions, which are specific for each protein (e.g. subcellular localization) or each residue within a protein (e.g. metal or disulfide bonding state).

To overcome this issue, we make use of grounding-specific Markov Logic Networks (gs-MLN), introduced in Lippi *et al.*[12], an extension that adds the ability of specifying per-grounding weights. The idea is to substitute the fixed per-formula weight w with a new function *ω* that depends on the particular grounding. The conditional distribution is modified to be of the form:

$$p(y|x;\theta )=\frac{1}{Z(x)}\text{exp}\underset{{\text{F}}_{i}\in \text{KB}}{\sum }\underset{\text{g}\in \text{G}({\text{F}}_{i})}{\sum }\omega (\text{g},\theta_{i})n_{\text{ij}}(x,y)$$

Here the variable g ranges over all satisfied groundings of formula F_*i*_, and the function *ω* evaluates the weight of the given grounding g according to a set of per-formula parameters *θ*_*i*_.

## Competing interests

The authors declare that they have no competing interests.

## Authors’ contributions

ST implemented the framework and ran the experiments. AP provided the original idea and supervised the experiments. ST and AP both wrote and revised the manuscript. Both authors read and approved the final manuscript.
